# Effectiveness of low-intensity pulsed ultrasound on osteoarthritis: molecular mechanism and tissue engineering

**DOI:** 10.3389/fmed.2024.1292473

**Published:** 2024-04-17

**Authors:** Jing Zhou, Eryu Ning, Lingfeng Lu, Huili Zhang, Xing Yang, Yuefeng Hao

**Affiliations:** ^1^Orthopedics and Sports Medicine Center, The Affiliated Suzhou Hospital of Nanjing Medical University, Suzhou, China; ^2^Gusu School, Nanjing Medical University, Suzhou, China

**Keywords:** low-intensity pulsed ultrasound, osteoarthritis, therapeutic potential, molecular mechanisms, tissue regeneration

## Abstract

Osteoarthritis (OA) is distinguished by pathological alterations in the synovial membrane, articular cartilage, and subchondral bone, resulting in physical symptoms such as pain, deformity, and impaired mobility. Numerous research studies have validated the effectiveness of low-intensity pulsed ultrasound (LIPUS) in OA treatment. The periodic mechanical waves generated by LIPUS can mitigate cellular ischemia and hypoxia, induce vibration and collision, produce notable thermal and non-thermal effects, alter cellular metabolism, expedite tissue repair, improve nutrient delivery, and accelerate the healing process of damaged tissues. The efficacy and specific mechanism of LIPUS is currently under investigation. This review provides an overview of LIPUS’s potential role in the treatment of OA, considering various perspectives such as the synovial membrane, cartilage, subchondral bone, and tissue engineering. It aims to facilitate interdisciplinary scientific research and further exploration of LIPUS as a complementary technique to existing methods or surgery. Ongoing research is focused on determining the optimal dosage, frequency, timing, and treatment strategy of LIPUS for OA. Additional research is required to clarify the precise mechanism of action and potential impacts on cellular, animal, and human systems prior to its integration into therapeutic applications.

## 1 Introduction

Osteoarthritis (OA) is the most prevalent degenerative joint disease affecting the elderly population, imposing a substantial burden on patients, families, and society. The primary risk factors comprise advanced age, obesity, female gender, genetic predisposition, and severe joint injuries (1). OA not only induces joint inflammation, pain, stiffness, and impaired function, but in advanced cases, it can also lead to muscle wasting, joint malformation, and ultimately, disability. Additionally, it can result in a cascade of complications, such as cardiovascular events, deep vein thrombosis in the lower extremities, hip fractures, and all-cause mortality (2). Consequently, the development of effective prevention and treatment strategies for OA has become a crucial challenge in the field of public health management.

Currently, conservative management of OA predominantly involves the use of symptomatic therapies, such as anti-inflammatory analgesics and cartilage-supporting nutrition. Although these interventions may provide temporary relief from pain, they are unable to arrest or reverse the progression of OA. Extended pharmacological treatment plans can result in negative side effects such as gastrointestinal issues, potential kidney damage, and the risk of developing a dependency on medication (3, 4). In addition, joint replacement surgery is frequently utilized to address advanced OA, despite its invasive nature, financially burdensome, likelihood of complications, and the finite durability of artificial joint prostheses. Despite years of research, the etiology of this prevalent and complex disease remains elusive, and there is a notable absence of efficacious treatments. Consequently, there is a pressing need for comprehensive exploration of a secure and efficient supplementary approach for managing OA. Since receiving initial approval from the US Food and Drug Administration (FDA) in 1994 for fracture treatment, therapeutic ultrasound has been acknowledged as a non-invasive, safe and non-carcinogenic modality in the management of musculoskeletal disease (5). This modality is known to enhance local circulation and lymphatic circulation, improve metabolism, promote tissue regeneration and nutrient status, and decrease muscle and connective tissue tension as well as sensory nerve excitability. Recently, low-intensity pulsed ultrasound (LIPUS) has emerged as a therapeutic modality for the treatment of OA. LIPUS is characterized by pulsed waves with frequencies ranging from 1 to 3 MHz and intensities below 1 w/cm^2^, making it a non-invasive physical stimulus for therapeutic purposes. Due to its low intensity and pulsed output mode, LIPUS exhibits minimal thermal effects, allowing for the targeted delivery of acoustic energy to tissues. LIPUS emits low intensity ultrasound in a pulsed wave mode, delivering mechanical energy to targeted tissue through high-frequency pressure waves, resulting in vibrations and collisions induced by the periodic acoustic waves. LIPUS has emerged as a promising therapeutic approach originally utilized for skeletal muscle disorders, demonstrating efficacy in enhancing fracture healing and promoting recovery from tendon and ligament injuries. Numerous research studies have demonstrated the significant impact of LIPUS on cellular metabolism and tissue repair in OA.

This review seeks to consolidate the existing body of research on the use of LIPUS in the treatment of OA over the past several decades, with a specific focus on application, outcomes and mechanisms of action on different affected tissues. The ultimate goal is to enhance the clinical effectiveness of LIPUS in the management of OA by elucidating its therapeutic principles and effects on synovial membranes, cartilage, and subchondral bones.

## 2 Definition and mechanism of LIPUS

### 2.1 Definition of LIPUS

**Figure 1:**
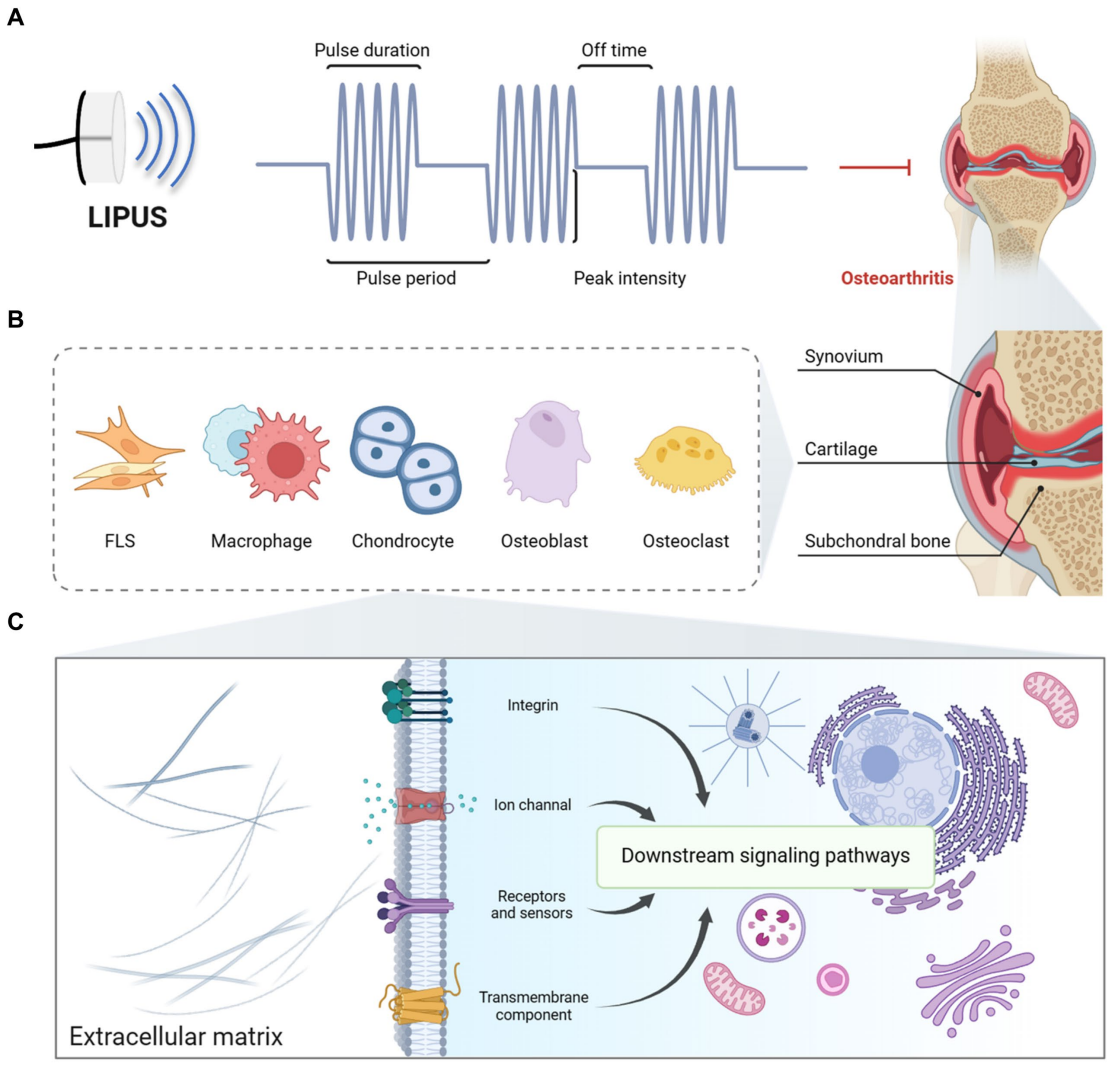
The typical features and potential mechanism of LIPUS. **(A)** Overall, the duty cycle of LIPUS in existing studies is 20%, the frequency ranges from 1 to 3 MHz, and the intensity ranges from 0.02 to 1 W/cm^2^. **(B)** LIPUS acts on FLSs, macrophages, chondrocytes, osteoblasts, and osteoclasts in the synovium, cartilage, and subchondral bone. **(C)** LIPUS facilitates the mediation of integrin, ion channel, and other cellular processes through mechano-biochemical signal transduction. This regulation subsequently influences downstream signaling pathways, ultimately impacting the pathophysiology of OA in the synovium, cartilage, and subchondral bone. Figures created with BioRender.com.

LIPUS is a therapeutic modality that utilizes ultrasonic pulsed waves to address various pathological conditions by leveraging mechanical, thermal, physical, chemical, and biochemical mechanisms, while preserving the integrity and viability of human tissues. For applications in both “*in vitro*” and non-invasive settings, LIPUS is subjected to sonication by diverse generators, transduced, and administered to the targeted treatment site. Currently, as shown in Figure 1A, LIPUS devices typically operate at frequencies of 1, 1.5, or 3 MHz, with some devices offering the option of 0.75 MHz for deeper lesions. LIPUS equipment commonly used today typically emits ultrasonic waves at a pulse rate of 1,000 Hz with a duty cycle of 20%, resulting in 200 μs of ultrasonic action time and 800 μs of intermittent time, and 1,000 cycles per second. The dosage of LIPUS can be subdivided into three categories: low dose (<1 W/cm^2^), medium dose (1–2 W/cm^2^), and high dose (2–3 W/cm^2^). In the majority of studies, LIPUS employs a dosage of 30–60 mW/cm^2^, a duty cycle of 20%, and a frequency of 1.5 MHz.

### 2.2 Cavitation and mechanical effects of LIPUS

LIPUS primarily operates via both thermal and non-thermal mechanisms. LIPUS emits periodic mechanical sound waves that penetrate tissues and cells, inducing vibration and collisions (6) (Figure 1B). By eliciting minimal thermal effects and substantial non-thermal effects within the targeted tissue, it modulates cellular metabolism, accelerates tissue healing processes, and ameliorates cellular ischemia and hypoxia, ultimately enhancing tissue nutrition and promoting tissue repair (7). LIPUS produces minimal thermal impact, as evidenced by a modest temperature of 0.5°C after a 10-min exposure in both *in vivo* and *ex vivo* conditions. The primary mechanisms of action for LIPUS are non-thermal in nature, specifically cavitation and mechanical effects, which have garnered significant attention from researchers.

#### 2.2.1 Cavitation effects of LIPUS

The ultrasonic cavitation effect mainly encompasses a sequence of dynamic events in which small gas-core cavitation bubbles undergo expansion and collapse in response to acoustic pressure generated by ultrasonic waves within the liquid medium. This phenomenon manifests in two distinct manifestations: stable and transient cavitation. Transient cavitation, which is predominantly utilized in the treatment of cancer and palliative care (8), involves the unstable oscillation of bubbles that expand to 2–3 times their resonance size before rapidly collapsing, releasing substantial energy that causes cellular damage and rapid tissue destruction. Both types of cavitation serve as the principal mechanisms responsible for ultrasound’s biological effects on targeted tissues. Nonetheless, the maintenance of a stable cavitation effect appears to be more efficacious in achieving favorable outcomes in ultrasound treatment.

#### 2.2.2 Mechanical effects of LIPUS

Mechanical effects mainly include acoustic flow and mass transfer enhancement. Acoustic flow pertains to the circulation that occurs around a liquid when a cavitation bubble vibrates and can be divided into macroscopic and microscopic flows. Macroscopic flow refers to the substantial movement of a fluid in alignment with the propagation of an ultrasound wave within a liquid. Comparatively, microscopic flows are characterized by a small vortices that form in close proximity to the point of oscillation. Furthermore, the mechanical impact of macroscopic flows is comparatively weaker than that of microscopic flows. Microscopic flows are a consequence of cavitation and have the potential to alter membrane permeability, influence diffusion ion rates, impact protein synthesis, and modulate cell secretion processes (9). In the realm of employing non-thermal effects in applications of LIPUS it is crucial to sustain stable cavitation and acoustic flows.

Improving mass transfer is a crucial component of the non-thermal effects of LIPUS. Ultrasound plays a key role in augmenting the movement of the fluid medium by increasing mass transfer and reaction rates. This phenomenon is particularly noticeable in cells and tissues treated with LIPUS, where it predominantly affects the cell membrane, cytosol, and boundary layers. The oscillation of bubbles creates micro-flows within the encompassing sound field, guiding reagents toward enzyme active sites or cells, thereby aiding in the release of biological substances into the medium and leading to discernible biological outcomes (10).

### 2.3 Conversion of mechanical signal into biochemical signaling

Acoustic signals elicit various interactions with tissues, including absorption, reflection, refraction, and transmission of mechanical pulse energy. Variations in acoustic impedance among tissues lead to differing absorption and propagation efficiencies of LIPUS, thereby impacting its therapeutic efficacy. LIPUS specifically targets tissues to induce a cavitation effect, facilitating enhanced acoustic flow and mass transfer, ultimately eliciting biophysical effects within the desired tissue. Currently, there is a lack of comprehensive understanding regarding the mechanisms by which tissues and cells perceive and respond to acoustic energy, leading to the translation of intercellular signals that regulate biological functions and reshape the microenvironment. Some researchers have sought to elucidate these biological processes through the framework of cellular mechanical transduction, as illustrated in Figure 1C. LIPUS, acting as a non-invasive form of mechanical stimulation, has the ability to dynamically modulate the cytoskeleton and induce extracellular matrix (ECM)-cytoplasm signal transduction by interacting with receptors such as integrins and ion channels located on the cell membrane. Subsequently, these signals are transmitted through the cytoskeleton to the nucleus, ultimately impacting gene expression and modifications, thereby initiating downstream cellular responses.

## 3 Mechanism of action of LIPUS on OA

### 3.1 Effects of LIPUS on synovium

The synovium plays a crucial role in the joint structure by working in conjunction with cartilage through an immune-inflammatory network, thereby contributing to the maintenance of joint cavity homeostasis (11). The various types of synoviocytes, such as fibroblast-like synoviocytes (FLSs), macrophages, and synovial stem cells, secrete synovial fluid, vesicles, and cytokines to facilitate lubrication, immunity and inflammation regulation, creating a specialized homeostatic microenvironment within the synovial membrane and joint cavity. Chronic low-grade synovial inflammation is present in all stages of OA and is closely associated with the clinical symptoms experienced by patients, including synovial hypertrophy, abnormal growth, vascular proliferation, and inflammatory reactions (12). The disruption of joint integrity during OA is attributed to an imbalance in the synovial inflammatory microenvironment, caused by the aberrant secretion of inflammatory factors within the synovium.

**Figure 2:**
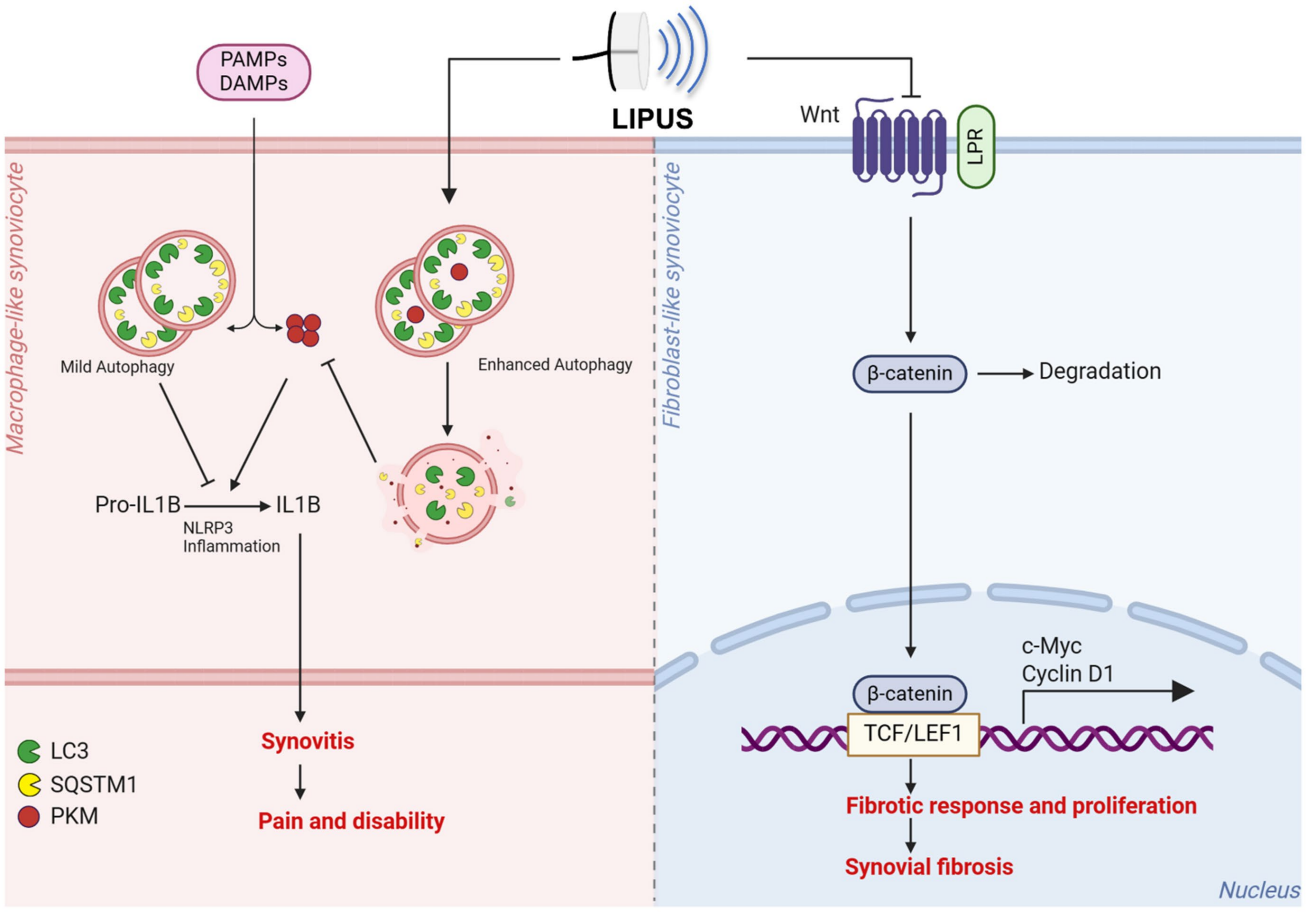
LIPUS holds potential as an effective approach to counteract synovial inflammation and fibrosis. LIPUS demonstrates the ability to mitigate synovial inflammation and joint dysfunction through the enhancement of autophagy in macrophage-like synoviocytes and the inhibition of mature IL-1B production. Additionally, LIPUS has the capacity to modulate the Wnt/β-catenin signaling pathway in fibroblast-like synoviocytes, facilitating β-catenin degradation and nuclear translocation, regulating downstream gene transcription, and ultimately impeding synovial fibrosis. Figures created with BioRender.com.

Recent research findings indicate that LIPUS may have the potential to serve as an efficacious intervention for mitigating synovial inflammation and fibrosis (Figure 2 and Table 1). Early implementation of LIPUS has been shown to delay the degradation of cartilage by decreasing synovial inflammation, suppressing MMP13 expression, and enhancing Col II expression within the cartilage (13). Several studies have demonstrated that LIPUS has the ability to suppress the formation of Toll-related receptor-myeloid differentiation factor in OA animal models and cell experiments, thereby controlling the synovial inflammatory response induced by lipopolysaccharide (LPS) (14). Additionally, LIPUS has been shown to effectively inhibit the proliferation, growth, and DNA cleavage of synoviocytes through the modulation of cytokines such as nitric oxide synthase (iNOS) and interleukin-1 beta (IL-1β). Moreover, in comparation to the control group, the LIPUS treatment group exhibited significantly reduced levels of inflammatory cell infiltration, synovial hyperplasia, pannus formation, and cartilage destruction, ultimately delaying the degeneration of OA articular cartilage (15). Furthermore, LIPUS was found to enhance the synthesis of hyaluronan (HA) and suppress the expression of hyaluronidase (HYAL) 2 HYAL2, resulting in the accumulation of high molecular weight HA (16). Nakamura et al. (17) have demonstrated that LIPUS intervention could effectively reduce knee tissue damage and decrease the levels of COX-2 positive cells in the knee compared to a control group in MRL/lpr mice. Additionally, LIPUS has been found to upregulate the phosphorylation of focal adhesion kinase (FAK) in synoviocytes and inhibit FAK phosphorylation to downregulate ERK, JNK, and p38 phosphorylation. These results suggest that LIPUS may regulate synoviocyte apoptosis and survival via the integrin/FAK/MAPK pathway (18).

**Table 1:** The effects of LIPUS on synovium.

PMID	Author (Year)	*In vitro* study & LIPUS dosage	*In vivo* study & LIPUS dosage	Main outcomes	
20,571,855	Nakamura et al. (2010)	HIG-82 cell line. 30 mW/cm^2^, 3 MHz for 15 min.	N/A	LIPUS down-regulated COX-2 and PGE2 and upregulated HAS2 and HAS3, promoting the anti-inflammatory system.	(16)
21,938,555	Nakamura et al. (2011)	HIG-82 cell line. 30 mW/cm^2^, 3 MHz for 15 min.	Male MRL/*lpr* mice, 30 mW/cm^2^, 1 MHz for 15 min/d for 7, 14, and 21d.	LIPUS suppressed the proliferation and growth of HIG-82 cells and reduced COX-2 and synovial hyperplasia *in vivo*.	(17)
25,096,496	Sato et al. (2014)	HIG-82 cell line. 30 mW/cm^2^, 3 MHz for 20 min.	N/A	LIPUS increased phosphorylation of Integrin β1, FAK, JNK, ERK, and p38.	(18)
30,262,135	Hsieh et al. (2018)	N/A	Male SD rats ACLT&M OA model. 0.1 W/cm^2^, 1.0 MHz for 4 wk.	LIPUS delayed cartilage degradation by reducing synovial inflammation and MMP 13 expression and enhancing Col II expression in cartilage.	(13)
31,500,508	Zhang et al. (2020)	TIB-202 and TIB-71 cell line. 30 mW/cm^2^, 1.5 MHz for 20 min.	Male C57BL/6 mice DMM-OA and air pouch model. 30 mW/cm^2^, 1.5 MHz for 20 min/d, 6 d/wk. for 2wk.	LIPUS ameliorated the gait patterns and synovial inflammation, which may be related to LIPUS-inhibitory mature IL1B production. LIPUS decreased the production of mature IL1B mainly through enhancing autophagy-mediated degradation of PKM and SQSTM1 in macrophages.	(22)
33,423,862	Feltham et al. (2021)	N/A	Dawley rats IAF-PTOA model. 30 mW/cm^2^, 1.5 MHz for 20 min/d for 2wk.	LIPUS treatment reduced CD68+ macrophages in the synovium and down-regulated IL-1β in the joint fluid.	(21)
34,611,513	Liao et al. (2021)	OA patients FLSs. 30 mW/cm^2^, 1.5 MHz for 20 min.	Male C57BL/6 mice DMM-OA model. 30 mW/cm^2^, 1.5 MHz for 20 min/d, for 7 and 14d.	LIPUS inhibited the proliferation and fibrotic response of FLSs through the Wnt/β-catenin pathway, thereby alleviating synovial fibrosis in OA	(19)

COX-2, cyclooxygenase-1; PGE2, prostaglandin E2; HAS, hyaluronan synthase; FAK, focal adhesion kinase; JNK, Jun N-terminal kinase; ERK, extracellular regulated protein kinase; ACLT&M, anterior cruciate ligament transection combined with meniscectomy; MMP, matrix metallopeptidase; Col II, collagen type II; IL, interleukin; PKM, pyruvate kinase; SQSTM1, sequestosome1; IAF-PTOA, intra-articular fracture post-traumatic osteoarthritis; FLSs, fibroblast-like synoviocytes; DMM, destabilization of the medial meniscus; Wnt, Wingless-Type MMTV Integration Site Family.

FLSs are essential mesenchymal components in synovial tissue and their dysregulated activation and resulting alterations in biological functions are significant contributors to OA pathogenesis. *In vitro* studies have shown that LIPUS can mitigate TGF-β1-induced fibrotic reactions and FLS proliferation. Furthermore, LIPUS has been found to suppress the Wnt/β-catenin signaling pathway and its associated downstream proteins in the synovial tissue of destabilization of the medial meniscus (DMM) mouse model. Collectively, the combined effects of this dual mechanism effectively inhibit synovial fibrosis and the downstream proteins associated with the Wnt/β-catenin pathway (19). Macrophages are identified as a crucial cell type within the synovium, playing a central role in the pathogenesis of OA. Studies have demonstrated a high concentration of activated macrophages in the affected joints of OA patients, which release pro-inflammatory factors that contribute to the acceleration of disease progression. The intervention of LIPUS has been shown to decrease the recruitment of macrophages. Following a 2-day treatment with LIPUS, it was observed that while the number of M1 macrophages decreased in the injury model, the number of M2 macrophages increased (20). This suggests that LIPUS plays a role in influencing on macrophage polarization, promoting the transition toward anti-inflammatory M2 macrophages and consequently reducing inflammatory responses in the vicinity of the joints. After a 4-week LIPUS intervention in a rat model of post-traumatic arthritis affecting the knee joint, there was a decrease in leukocyte infiltration within the synovium. Furthermore, LIPUS stimulation resulted in a significant reduction in the number of CD68+ macrophages and restricted their localization within the subintimal synovium (21). Moreover, LIPUS was found to enhance autophagy levels and expedite the formation of the SQSTM1 (sequestosome1)-PKM (pyruvate kinase, muscle) complex in macrophages treated with LPS-ATP. Through the regulation of SQSTM1-dependent autophagic degradation of muscle-type pyruvate kinase 2 in macrophages, LIPUS effectively suppresses the production of mature IL-1β, consequently improving synovial inflammation and gait performance in animal models (22).

### 3.2 Effects of LIPUS on cartilage

**Figure 3:**
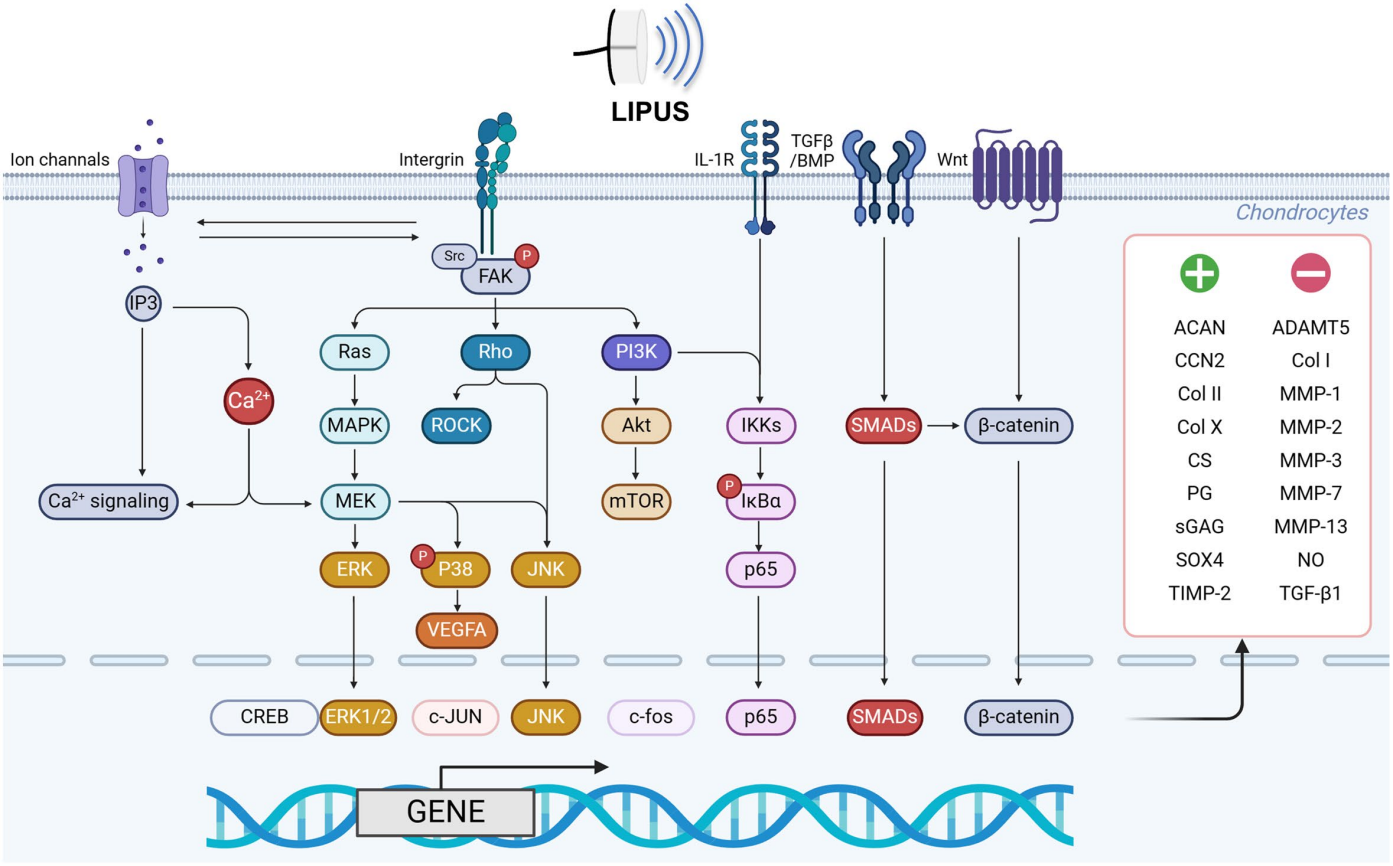
LIPUS regulates chondrocyte function via multiple pathways. LIPUS exerts regulatory effects on chondrocyte proliferation, differentiation, and metabolism through modulation of ion channels and integrin-related FAK/PI3K signaling pathways. Specifically, the activation of ion channels facilitates calcium influx, cytoskeletal regulation, and MEK/ERK pathway activation. Additionally, LIPUS suppresses MMP13 and p38 phosphorylation in healthy chondrocytes by activating the mechanosensitive integrin/FAK pathway and modulating the Ras/MEK/ERK, Rho/ROCK, and FAK/PI3K signaling cascades. In OA chondrocytes, LIPUS may impede chondrocyte proliferation and differentiation by inhibiting p38 phosphorylation. Furthermore, LIPUS modulates the catabolic and inflammatory effects of IL-1β by inhibiting the binding of IL-1β to the IL-1β receptor complex and the downstream NF-κB pathway. Conversely, LIPUS was found to regulate TGF-β/BMP receptors on the chondrocyte membrane, activating the SMADs pathway and influencing the downstream Wnt/β-catenin signaling pathway to exert its biological regulatory functions. Figures created with BioRender.com.

Degeneration of articular cartilage is a key pathological feature of OA and plays a pivotal role in the diagnosis of OA. Multiple biological factors, such as inflammation and mechanical forces, can disrupt the balance of chondrocytes, leading to decreased synthesis of the ECM of cartilage and diminished resistance of articular cartilage to stress, ultimately causing injury (Figure 3 and Table 2).

**Table 2:** The effects of LIPUS on cartilage.

PMID	Author (Year)	*In vitro* study & LIPUS dosage	*In vivo* study & LIPUS dosage	Main outcomes	
11,603,707	Cook et al. (2001)	N/A	Male New Zealand White rabbits critical-sized osteochondral defect OA model. 30 mW/cm^2^, 1.5 MHz, 20 min/d for 4, 8, 12, 24, and 52 wk.	LIPUS improved the morphologic features and histologic characteristics of the repaired cartilage. Better repair at earlier times. A treatment time of 40 min/d increased the histologic quality.	(59)
11,745,554	Nishikori et al. (2002)	Human primary chondrocytes, 30 mW/cm^2^, 1.5 MHz, 20 min/d for 3 wk.	N/A	LIPUS promoted the synthesis of CS, especially C6S, although it did not significantly enhance cell number and stiffness.	(60)
12,498,950	Zhang et al. (2002)	White Leghorn chick embryos sterna explants. 30 mW/cm^2^, 1.5 MHz, 20 min/d for 1, 3, 5, and 7d.	N/A	LIPUS increased matrix production. LIPUS increased Col X in certain regions of the sternum. LIPUS stimulated bone formation by increasing hypertrophy of chondrocytes directed to terminal differentiation.	(61)
14,654,159	Zhang et al. (2003)	White Leghorn chick embryos sterna chondrocytes. 2 and 30 mW/cm^2^, 1.5 MHz, 20 min/d for 1, 3, 5, and 7d.	N/A	LIPUS did not affect chondrocyte viability and proliferation. LIPUS acted transiently to decrease the expression of ECM-related genes, followed by up-regulation of Col II and ACAN. LIPUS slowed the progression of chondrocyte hypertrophic differentiation.	(62)
14,689,492	Duda et al. (2004)	N/A	Hyaline-like cartilage specimens were generated *in vitro* and subcutaneously implanted in the backs of female homozygotic athymic nude mice, 30 mW/cm^2^, 1.5 MHz, 20 min/d for 1, 3, 6, and 12 wk.	LIPUS increased neocartilage formation. The mechanical stability of the neocartilage specimens increased with treatment time and reached values of native cartilage.	(63)
15,896,276	Jia et al. (2005)	N/A	Male New Zealand White rabbits bilateral full-thickness osteochondral defect OA model, 30 mW/cm^2^, 1.5 MHz for 8 wk.	LIPUS increased the scores of the gross appearance grades, histological grades, and the optical density of toluidine blue in the tissues.	(64)
18,359,144	Tien et al. (2008)	Young children’s primary chondrocytes. 18, 48, 72, and 98 mW/cm^2^, 1.0 MHz, 20 min/d for 14d.	N/A	LIPUS increased ACAN synthesis in a time-dependent manner. LIPUS revealed no significant influence on cell proliferation. Human chondrocytes harvested from older donors become less responsive to LIPUS.	(65)
18,483,198	Cook et al. (2008)	N/A	Male canine osteochondral plugs OA model. 30 mW/cm^2^, 1.5 MHz, 20 min/d for 6 and 12 wk.	LIPUS improved the interface repair tissue, which had a more normal translucent appearance. LIPUS improved the cell morphologic characteristics of the interface repair tissue and increased subchondral bone regeneration.	(36)
18,853,213	Korstjens et al. (2008)	Human primary chondrocytes and cartilage explants. 30 mW/cm^2^, 1.5 MHz, 20 min/d for 6 d.	N/A	LIPUS stimulated chondrocyte proliferation and matrix production in human articular cartilage chondrocytes *in vitro*.	(66)
19,810,106	Naito et al. (2010)	N/A	Male SD rats ACLT+MMx OA model. 30 mW/cm^2^, 1.5 MHz, 20 min/d for 7, 14, and 28 d.	LIPUS increased Col II via the activation of chondrocytes and induction of Col II mRNA expression, thereby exhibiting chondroprotective action.	(67)
20,175,971	Gurkan et al. (2010)	N/A	Male Hartley guinea pigs joint immobilization OA model. 30 mW/cm^2^, 1.5 MHz, 20 min/d for 2, 3, 4, 6, 10, 12, 15, and 18 months.	LIPUS did not fully prevent cartilage degeneration but diminished the severity of the disease. LIPUS decreased the degree of TGF-β1 production.	(68)
20,938,751	Vaughan et al. (2005)	Steer primary chondrocytes. 30, 100, 200, 300 mW/cm^2^, 1.5 MHz, 20 min/d for 5, 10, 15, and 20 d.	N/A	LIPUS (30 mW/cm^2^) had no net effect on the synthesis of sGAG by adult bovine articular chondrocytes. LIPUS (100 mW/cm^2^) induced an initial transient up-regulation of SO4 incorporation and sGAG content in agarose constructs.	(69)
21,567,132	Li et al. (2011)	N/A	Male/female New Zealand white healthy rabbits ACLT OA model. 40 mW/cm^2^, 3 MHz, 20 min/d, 6d/wk. for 6 wk.	The early application of LIPUS could delay the degeneration of articular cartilage. This effect was related to the decreased expression of MMP-13 and suppression of ERK1/2, p38 signaling.	(70)
22,920,551	Ito et al. (2012)	Wistar rat primary chondrocytes. 7.5, 30, and 120 mW/cm^2^, 1.5 MHz for 1 or 3 h.	N/A	LIPUS inhibited the induction of MMP13 mRNA expression induced by IL-1β in an intensity-dependent manner.	(71)
23,646,806	Li et al. (2013)	N/A	New Zealand white healthy rabbits ACLT OA model. 40 mW/cm^2^, 3 MHz, 20 min/d, 6d/wk. for 4 wk.	LIPUS decreased the Mankin scores in articular cartilage and inhibited MMP-13 expressions to a large extent through p38, ERK1/2 and JNK pathways.	(72)
24,507,771	Yang et al. (2014)	N/A	Female Japanese white rabbits critical-sized osteochondral defect OA model. 30 mW/cm^2^, 1.5 MHz, 20 min/d for 3 months.	LIPUS did not cause Col II deposition and chondrocyte proliferation.	(73)
24,612,644	Jang et al. (2014)	Bovine stifle joints, osteochondral explants and CPC. 36.7 mW/cm^2^, 1 MHz, for 7 d or 27.5 mW/cm^2^, 3.5 MHz, for 5 min.	N/A	LIPUS-induced CPC migration was blocked by suppressing FAK phosphorylation with an SFK inhibitor that blocks FAK phosphorylation.	(74)
24,742,749	Cheng et al. (2014)	Rabbit primary chondrocytes. 40 mW/cm^2^, 3 MHz, 20 min/d for 6 d.	Male New Zealand white rabbits ACLT OA model. 40 mW/cm^2^, 3 MHz, 20 min/d for 6 d.	LIPUS decreased the degradation of Col II and ACAN, inhibited MMP-1&MMP-13 and activated the integrin-FAK-PI3K/Akt mechanochemical transduction pathway.	(75)
25,571,661	Xu et al. (2014)	N/A	Female rabbit Hulth OA model. 50 ± 10%mW/cm^2^, 800 ± 5% kHz, 20 min/d for 2, 4, and 8 wk.	LIPUS repaired the damaged cartilage by reducing the expression of MMP-3, 7, 13, inhibiting NO secretion and promoting the synthesis of Col II and proteoglycan in cartilage.	(76)
25,267,432	Tan et al. (2015)	Human and pig cartilage explants. 30 mW/cm^2^, 1.5 MHz, 20 min/d for 4 wk.	N/A	LIPUS reduced the expression of the ACAN and Col II genes. LIPUS prevented degenerative changes in pig knee cartilage explants and reduced degeneration in human cartilage samples.	(77)
25,736,607	Xia et al. (2015)	Rabbit primary chondrocytes. 20, 30, 40 and 50 mW/cm^2^, 3 MHz, 20 min/d for 6 d.	Male New Zealand white rabbits ACLT OA model. 20, 30, 40 and 50 mW/cm^2^, 3 MHz, 20 min/d for 6 d.	LIPUS affected the expression of Col II and MMP13 through the integrin–p38 MAPK signaling pathway.	(31)
25,915,185	Ting et al. (2015)	Bovine primary chondrocytes. 55 mW/cm^2^, 1 MHz, 20 min/d for 10 d.	N/A	LIPUS increased sGAG and collagen protein production along with a higher Young’s Modulus. LIPUS upregulated gene expression for Col I, Col X, MMP-1, MMP-2, MMP-13, and p-p38.	(23)
26,396,170	Xia et al. (2015)	N/A	Male New Zealand white Rabbits ACLT OA model. 40 mW/cm^2^, 3 MHz, 20 min/d, 6d/wk. for 6wk.	LIPUS protects cartilage from damage in early-stage osteoarthritis via the integrin/FAK/MAPK pathway.	(78)
26,706,677	Ji et al. (2015)	Rabbit primary chondrocytes. 40 mW/cm^2^, 3 MHz, 20 min/d for 7d.	Male New Zealand rabbits inner patellar ligament defect OA model. 40 mW/cm^2^, 3 MHz, 20 min/d for 7 d.	LIPUS increased the expression of serum TIMP-2 in the OA model group and decreased MMP-13.	(79)
27,774,951	Du et al. (2016)	Human primary chondrocytes. 30 mW/cm^2^, 1.5 MHz, 20 min/d for 7d.	N/A	LIPUS increased the expressions of Col II and ACAN and reduced MMP-13 via PI3K/Akt pathway.	(80)
27,364,595	Uddin et al. (2016)	Human cartilage explants, C-28/I2 and C3H10T1/2 cell lines. 30 mW/cm^2^, 1 MHz, 20 min/d for 7d.	N/A	LIPUS increased the PG content in human cartilage explants and inhibited IL-1β-induced loss of proteoglycans. LIPUS increased rates of chondrocyte migration and proliferation and promoted chondrogenesis in MSCs. LIPUS suppressed IL-1β-induced activation of p-p65 and p-IκBα, leading to reduced expression of MMP13 and ADAMT5 in chondrocytes.	(30)
27,729,291	Nishida et al. (2017)	HCS-2/8 cell line, rat primary epiphyseal and articular cartilage cells, and CCN2-deficient chondrocytes. 60 mW/cm^2^, 3 MHz for 30 min, 1 h, and 5 h.	N/A	LIPUS-stimulated Ca2+ influx activated chondrocyte differentiation represented by CCN2 production was mediated via MAPK pathways, which in turn was supported by the induced CCN2 molecules in articular chondrocytes.	(24)
28,638,681	Yılmaz et al. (2017)	N/A	Male SD rats MIA-OA model. 40 mW/cm^2^, 3 MHz, 20 min/d for 15 d.	LIPUS had systemic proliferative and regenerative effects on cartilage and tissue.	(44)
29,111,161	Zahoor et al. (2018)	N/A	Male SD rats IAF-PTOA model. 30 mW/cm^2^, 1.5 MHz, 20 min/d, 5d/wk. for 2wk.	LIPUS improved the gait and PTOA pathology of the animals. LIPUS applied at the early stage of IAF or during PTOA development had lasting effects on the PTOA pathology in rat knees.	(81)
29,762,847	Tang et al. (2018)	N/A	Male/female New Zealand rabbits critical-sized osteochondral defect OA model. 30.0 ± 5.0 mW/cm^2^, 1.5 MHz, 20 min/d for 4 or 8 wk.	LIPUS and FGF2 combination promoted the synthesis, secretion of collagen in chondrocytes and the differentiation and maturation of chondrocytes during the repair of cartilage defects.	(82)
30,348,521	Sekino et al. (2018)	ATDC5 cell line. 30 and 60 mW/cm^2^, 1.5 MHz, 20 min/d for 3, 5, and 7d.	N/A	LIPUS induced collagen synthesis and the remodeling of ACAN via the activation of ERK1/2.	(33)
30,262,135	Hsieh et al. (2018)	N/A	Male SD rats ACLT+MMx OA model. 0.1 W/cm^2^, 1.0 MHz, 20 min/d for 4 wk.	LIPUS reduced Mankin scores, inflammatory cells and MMP 13 expression and increased Col II expression in rats with PTOA.	(13)
30,322,672	Li et al. (2019)	N/A	Female SD rats ACLT OA model. 30 mW/cm^2^, 3 MHz, 20 min/d, 5d/wk. for 6 wk.	LIPUS improved cartilage degeneration and subchondral sclerosis during OA progression.	(46)
30,659,392	Pan et al. (2019)	Rabbit primary chondrocytes. 0.126, 0.157, or 0.25 MPa, 1 MHz, 20 min/d for 7 d.	N/A	LIPUS plus CCO promoted ECM deposition by accelerating the TGF-β/Smad-signaling pathway in chondrocytes.	(83)
31,975,545	Guan et al. (2020)	Mouse primary chondrocytes. 30 mW/cm^2^, 1.5 MHz for 20 min.	Male C57BL/6 J mice DMM OA model. 30 mW/cm^2^, 1.5 MHz, 20 min/d for 2 wk.	LIPUS ameliorated VEGFA-mediated disorders in cartilage ECM metabolism and chondrocyte hypertrophy during OA development.	(34)
33,175,286	Vahedi et al. (2021)	N/A	Male Bergamasca–Massese sheep critical-sized osteochondral defect OA model. 200 mW/cm^2^, 20 min/d for 2 months.	LIPUS induced chondrogenesis by enhancing the proteoglycans, expression of cartilage markers and stimulating the chondrocytes to produce ECM proteins.	(84)
32,281,401	Sang et al. (2021)	C28/I2 and CHON-001 cell line. 50 or 100 mW/cm^2^, 1.5 MHz for 24, 48, 72, and 96 h.	Male C57BL/6 mice ACLT OA model. 40 mW/cm^2^, 3 MHz, 20 min/d for 8 wk.	LIPUS improved the arthritis score and weight-bearing abilities. LIPUS reduced IL-6, IL-8 and TNF-α levels in the synovial fluid of OA mice. LIPUS promoted chondrocyte proliferation and differentiation by activating FAK signaling.	(27)
35,184,911	Tavakoli et al. (2022)	N/A	Male Dunkin Hartley guinea pigs MIA OA model.	LIPUS was superior to PRP in improving the mechanical properties of the articular cartilage, while LIPUS and PRP injection effectively improved joint lubrication.	(85)
35,912,499	Sabanci et al. (2022)	CHON-001 cell line. 0.5 W/cm^2^, 3 MHz for 2.5, 5, 7.5, and 10 min.	N/A	LIPUS induced cartilage cell proliferation; however, no positive effect was observed on cartilage cell migration.	(86)
37,870,591	Kojima et al. (2023)	N/A	Female ICR mice MIA OA model. 30 mW/cm^2^, 2 MHz, 20 min/d for 1 or 4 wk.	LIPUS attenuated cartilage degeneration in early OA by relieving inflammation and enhancing the inhibitory effect of lubricin on cartilage degeneration.	(35)
38,517,601	Wu et al. (2024)	Human and mouse primary chondrocytes. 30 mW/cm^2^, 1.5 MHz for 20 min.	C57BL/6 J mice ACLT + PM OA model. 30 mW/cm^2^, 2 MHz, 20 min/d for 4 wk.	LIPUS exerted a active effect on OA by activating TRPV4, inducing calcium inward flow, and facilitating the entry of NF-κB into the nucleus to regulate synthetic matrix gene transcription.	(37)
38,493,143	Pan et al. (2024)	Rat primary chondrocytes. 30, 45, and 60 mW/cm^2^, 1.5 MHz for 10, 20, and 30 min.	Male SD rats DMM OA model. 30 mW/cm^2^, 1.5 MHz, 20 min/d, 5d/wk. for 6wk.	LIPUS decreased the expression of YAP by restoring the impaired autophagy capacity and inhibiting the binding between YAP and RIPK1, thereby delaying the progression of OA.	(41)

CS, chondroitin sulfate; Col X, collagen type X; Col II, collagen type II; ACAN, aggrecan; ACLT + MMx, anterior cruciate/medial collateral ligament transection and medial meniscus resection; TGF-β1, transforming growth factor-β1; sGAG, sulphated glycosaminoglycan; MMP, matrix metallopeptidase; ERK, extracellular signal regulated kinases; JNK, Jun N-terminal kinase; CPC, chondrogenic progenitor cell; FAK, focal adhesion kinase; SFK, src family kinase; PI3K, phosphatidylinositol 3-kinase; Akt (PKB), protein kinase B; NO, nitrous oxide; MAPK, mitogen-activated protein kinases; TIMP-2, tissue inhibitor of metalloproteinase-2; MSC, mesenchymal stem cell; PG, proteoglycan; IL, interleukin; IκBα, inhibitor kappa B alpha; ADAMT5, a disintegrin and metalloproteinase with thrombospondin motif 5; CCN2, CCN family protein 2; MIA, monosodium Iodoacetate; IAF-PTOA, intra-articular fracture post-traumatic osteoarthritis; FGF2, fibroblast growth factors; CCO, clematis chinensis osbeck; ECM, extracellular matrix; DMM, destabilization of the medial meniscus; VEGFA, vascular endothelium growth factor A; TNF-α, tumor necrosis factor; PRP, platelet-rich plasma. TRPV4, transient receptor potential channel 4; YAP, Yes-associated protein; RIPK1, receptor-interacting protein kinase 1.

Cartilage tissue demonstrates sensitivity to mechanical stimuli, with LIPUS having the potential to influence the proliferation and differentiation of chondrocytes. Studies have shown that LIPUS can transmit sustained wave energy and modulate the increased expression of TGF-β, thereby promoting the proliferation of bovine chondrocytes and the upregulation of cartilage-related markers such as MMP-1 and Col I (23). Additionally, research has indicated (24) that the CCN protein family 2 may play a role in enhancing the proliferation and differentiation of articular cartilage and osteoblasts affected by OA, thereby potentially supporting the proliferation of injured articular cartilage. Khanna et al. (25) conducted experiments using rabbit and dog models with articular cartilage defects, demonstrating that LIPUS significantly improved cartilage repair, morphological changes, and chondrocyte proliferation. In a separate study (26), LIPUS was applied to stimulate articular chondrocytes in OA rat models, resulting in the synthesis of chondrocyte DNA, upregulation of the ERK signaling pathway and β1 integrin expression, and enhanced chondrocyte proliferation and differentiation through the activation of focal adhesion kinase (FaK) signaling. The inhibition of FaK has been shown to impede lipase-mediated cell proliferation and differentiation *in vitro*, as well as reduce inflammation in OA mice (27). Xie et al. (28) further confirmed that LIPUS enhanced the proliferation of BMSC through the regulation of phosphoinositide 3-kinase (PI3K)/protein kinase B (AKt) pathway proteins and the upregulation of cyclin D1 expression. Adipose-derived stem cells also exhibited differentiation into chondrocytes following 2 weeks of LIPUS stimulation. These collective findings suggest that LIPUS may have the potential to delay OA progression by promoting chondrocyte proliferation in injured joints and accelerating soft tissue repair.

In the realm of OA cartilage metabolism, the degradation of joint tissue occurs as a result of a imbalance between catabolic and anabolic processes. Specifically, there is a notable increase in ECM degradation of OA articular cartilage, with MMPs playing a pivotal role in this process (29). Studies have shown that LIPUS stimulation of OA articular cartilage can attenuate the expression of catabolic enzymes, such as MMP-13, and the platelet response protein disintegrant and metallopeptidase 5 in chondrocytes (30). Moreover, research has demonstrated that ultrasound has the capability to initiate mechanoconductive cell signaling pathways, leading to the stimulation of chondrocytes for the production of Col II, integrin β1 (31), and proteoglycan, as well as mesenchymal stem cells to facilitate the restoration of damaged tissues in OA joints (32). LIPUS has been shown to promote collagen synthesis and aggrecan remodeling, thereby providing therapeutic benefits via the activation of ERK1/2, enhancement of hypertrophic chondrocytes, inhibition of endochondral osteogenesis, and induction of ECM regeneration (33). LIPUS can also inhibit disruptions in cartilage ECM metabolism and chondrocyte hypertrophy mediated by VEGF during OA progression by regulating VEGFA expression in OA chondrocytes and suppressing p38 MAPK activity (34). Kojima et al. (35) abserved a notable reduction in Osteoarthritis Research Society International (OARSI) scores was noted in the group treated with LIPUS irradiation. Conversely, the non-LIPUS group exhibited extensive areas of dual positivity for collagen II and denatured collagen detection reagent (DCDR), while the LIPUS group displayed a limited number of DCDR-positive areas. Furthermore, a significant decrease in macrophage numbers within the articular capsule was observed in the LIPUS irradiated group. Cook et al. (36) further established that extended LIPUS treatment duration enhanced joint function and facilitated joint function recovery in OA. Furthermore, LIPUS stimulation of OA cartilage led to a notable increase in Col II synthesis and mRNA expression, thereby expediting soft tissue repair. Wu et al. (37) indicated that LIPUS modulated primary cilia expression and enhanced synthetic matrix metabolism in articular chondrocytes, with a concomitant association with primary cilia. Furthermore, LIPUS demonstrated a significant impact on OA by activating Transient Receptor Potential Vanilloid 4 (TRPV4), prompting calcium influx, and facilitating nuclear translocation of NF-κB to regulate gene transcription related to synthetic matrix production. Inhibition of TRPV4 influenced primary cilia expression following LIPUS stimulation, while depletion of primary cilia similarly affected TRPV4 activity.

Tanaka et al. (38) showed that the level of autophagy is modified in OA-affected cartilages, with a decrease in cartilage damage and an increase in the expression levels of chondrocyte autophagy markers Beclin-1, ATG7, fibroblast growth factor 18 and fibroblast growth factor receptor 4 observed under the influence of LIPUS. LIPUS was found to stimulate the release of MSC exosomes through the activation of autophagy, thereby augmenting the beneficial impact of MSCs on OA cartilage by inhibiting GW4869, a known exosome release inhibitor (39). In a study by Wang et al. (40), it was shown that LIPUS had a discernible influence on autophagy in chondrocytes, leading to the conclusion that autophagy activation could facilitate the chondrogenic differentiation of mesenchymal stem cells. LIPUS was also found to reduce the expression of Yes-associated protein (YAP) through restoration of impaired autophagy capacity and inhibition of the interaction between YAP and receptor-interacting protein kinase 1 (RIPK1), consequently leading to a delay in the advancement of OA. Results from animal experimentation demonstrated that LIPUS could effectively impede cartilage degeneration and mitigate the progression of OA (41).

In summary, LIPUS regulates chondrocyte proliferation, differentiation, autophagy levels, and the balance between synthesis and catabolism through complex signaling pathways to enhance the function of OA joints and promote recovery.

### 3.3 Effects of LIPUS on subchondral bone

During the early stages of OA, patients undergo subchondral bone loss, with progressive subchondral osteopenia developing as the disease advances. Subchondral sclerosis disrupts the normal mechanical distribution of loads within the joint, hindering the ability of the overlying cartilage to effectively cushion pressure. Research has demonstrated that osteoblasts play a crucial role in the inflammatory response and can produce chemokines that trigger inflammatory reactions (42) (Table 3). Given the sensitivity of osteoblasts to mechanical stimulation, the use of LIPUS has been considered an effective treatment for subchondral OA.

**Table 3:** Effects of LIPUS on subchondral bone.

PMID	Author (Year)	*In vitro* study & LIPUS dosage	*In vivo* study & LIPUS dosage	Main outcomes	
18,483,198	Cook et al. (2008)	N/A	Male canine osteochondral plugs OA model. 30 mW/cm^2^, 1.5 MHz, 20 min/d for 6 and 12 wk.	LIPUS improved the cell morphologic characteristics of the interface repair tissue and increased subchondral bone regeneration.	(36)
27,600,474	Yamaguchi et al. (2016)	N/A	Wistar rats osteochondral defect OA model. 30 mW/cm^2^, 1.5 MHz for 20 min/d, 5d/wk., 4 or 8 wk.	LIPUS irradiation may promote BV/TV reconstruction in the subchondral bone.	(43)
28,638,681	Yılmaz et al. (2017)	N/A	Male SD rats MIA delivered OA model. 40 mW/cm^2^, 3 MHz, 20 min/d for 15d.	LIPUS has systemic proliferative and regenerative effects on cartilage and subchondral bone BMD values.	(44)
30,322,672	Li et al. (2019)	N/A	Female SD rats ACLT OA model. 30 mW/cm^2^, 3 MHz, 20 min/d, 5d/wk. for 6 wk.	LIPUS improved cartilage degeneration and subchondral sclerosis during OA progression.	(46)
32,060,941	Yi et al. (2020)	N/A	Male/female New Zealand white rabbits Col II inducted OA model. 30 mW/cm^2^, 1 MHz for 20 min/d, 3 or 6 wk.	LIPUS improved the quality of subchondral bone by inhibiting osteoclast activity and the TGF-β1/Smad3 signaling pathway in subchondral bone.	(87)
33,774,341	Yi et al. (2021)	MC3T3-E1 cell line, 30 mW/cm^2^, 1 MHz for 20 min	Male/female New Zealand white rabbits Col II inducted OA model. 30 mW/cm^2^, 1 MHz for 20 min/d, 3 or 6 wk.	LIPUS suppressed IL-6 expression, mediated partly by suppressing the TGFβ1/Smad3 pathway *in vitro*.	(88)
38,161,708	Lee et al. (2023)	RAW 264.7 cell line. 30 mW/cm^2^, 3.3 MHz, 20 min/d for 7 days.	Male SD rats ACLT OA model. 0.1 W/cm^2^, 3.3 MHz, 20 min/d for 4 wk.	LIPUS involved the suppression of osteoclastogenesis and the alteration of DRG profiles.	(45)

BV/TV, bone volume per tissue volume; MIA, monosodium Iodoacetate; BMD, bone mineral density; ACLT, anterior cruciate ligament transection; TGF-β1, transforming growth factor-β1; IL, interleukin; DRG, dorsal root ganglia.

Yamaguchi et al. (43) conducted a study examining the synergistic effects of LIPUS and MSC therapy for OA, finding that the combined intervention result in greater improvements in cartilage repair scores and bone volume (BV)/tissue volume (TV) compared to their individual applications. Yılmaz et al. (44) developed a monoiodoacetate (MIA) rat model to investigate the systemic regenerative effects of LIPUS on cartilage and subchondral bone, demonstrating increased bone mineral density (BMD) values. Lee et al. (45) indicated that early intervention with LIPUS treatment can provide protective benefits against the advancement of OA, such as decreased tissue degradation, alleviated pain symptoms, enhanced subchondral bone microarchitecture, and reduced sensory innervation. Additionally, regular LIPUS therapy appears to inhibit osteoclastogenesis, potentially through its impact on sensory innervation suppression in OA. A separate study (46) investigated the effects of LIPUS on changes in articular cartilage and subchondral bone under both normal and functionally disused conditions. The study found that OA was associated with reduced cartilage thickness and glycosaminoglycan sulfate content, as well as increased thickness of the subchondral cortical bone plate and heightened subchondral trabecular bone density during periods regular joint use. Furthermore, the density and spatial distribution of chondrocytes exhibit an increase during the progression of OA, leading to alterations in both cartilage degradation and subchondral bone structure. These changes are associated with a decline in mechanical properties of osteoarthritic cartilage, which can be significantly alleviated by LIPUS treatment, particularly under conditions of regular joint loading. In summary, LIPUS contributes to mitigating cartilage degeneration and subchondral alterations during the progression of OA.

## 4 LIPUS and tissue engineering

**Figure 4:**
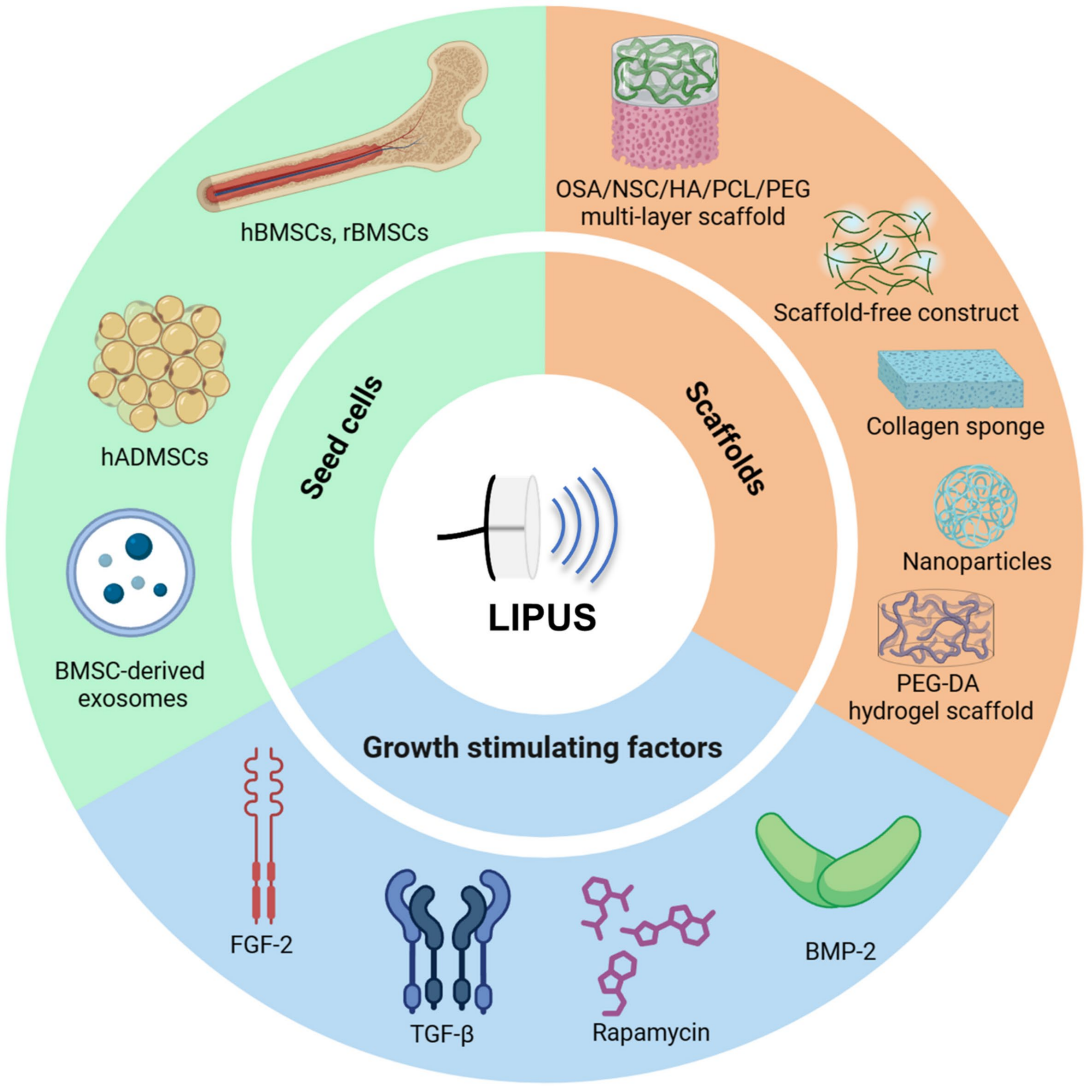
Tissue engineering, particularly with LIPUS, has witnessed significant advancements and is now extensively employed for joint repair. Researchers have identified bone marrow and adipose-derived mesenchymal stem cells along with their exosomes, induced their differentiation into specialized cells, developed diverse biocompatible scaffolds, incorporated various growth factors to enhance the synergistic effects of tissue engineering technology and LIPUS, ultimately facilitating OA tissue repair. Figures created with BioRender.com.

Stem cells and tissue engineering approaches are key areas of study in regenerative medicine, particularly in the context of regeneration. The combination of stem cells and tissue engineering techniques offers the potential to create cartilage tissue that closely resembles native hyaline cartilage. The essential components of artificial cartilage tissue engineering include seed cells, scaffolds, and growth factors. This review provides a comprehensive analysis of the application of LIPUS in cartilage tissue engineering, focusing on these critical elements (Figure 4 and Table 4).

**Table 4:** LIPUS and tissue engineering.

PMID	Author (Year)	*In vitro* study & LIPUS dosage	*In vivo* study & LIPUS dosage	Main outcomes	
16,912,415	Schumann et al. (2006)	hMSCs. 1, 7, 14 and 21 d.	N/A	Ultrasound-treated cell-scaffold constructs significantly increased chondrogenic marker gene expression and ECM deposition.	(89)
18,616,830	Takeuchi et al. (2008)	Pig primary chondrocytes. 30 mW/cm^2^, 1.5 MHz for 3, 7, 10 and 14 d.	N/A	LIPUS promoted the proliferation of chondrocytes and the production of Col IX in a 3D culture using a collagen sponge. The anabolic LIPUS signal transduction to the nucleus via the integrin/PI3K/Akt pathway rather than the integrin/MAPK pathway was generally associated with cell proliferation.	(50)
20,510,190	Lai et al. (2010)	hMSCs. 200 mW/cm^2^, 1 MHz, 20 min/d for 1, 2, 3, and 4wk.	N/A	LIPUS enhanced chondrogenic differentiation of hMSCs treated with TD. The combined treatment of LIPUS and TD induced integrin β1, Sox9, ACAN and Col II mRNA expression. LIPUS alone increased osteogenic differentiation. LIPUS with BMP-induced ALP and Runx2 mRNA expression.	(58)
21,116,901	Uenaka et al. (2010)	Rat primary chondrocytes. 30 mW/cm^2^, 1.5 MHz for 1, 7, 28, and 35d.	N/A	LIPUS enhanced matrix production in the construct, and its combination with the scaffold-free construct might become a feasible tool for the production of implantable constructs of better quality.	(51)
27,600,474	Yamaguchi et al. (2016)	N/A	Wistar rats osteochondral defect OA model. 30 mW/cm^2^, 1.5 MHz for 20 min/d, 5d/wk., 4 or 8 wk.	MSC injection improved the cartilage repair score, and LIPUS irradiation improved BV/TV. Combination treatment promoted both cartilage repair and BV/TV improvement.	(43)
27,883,051	Aliabouzar et al. (2016)	hBMSCs. 10, 30, 70, 100, 150 and 300 mW/cm^2^, 1.5 MHz, 3 min/d, for 1, 3, and 5d.	N/A	LIPUS combined with microbubbles enhanced GAG and Col II production.	(53)
29,064,570	Aliabouzar et al. (2018)	hBMSCs. 100 mW/cm^2^, 1.5 MHz, 3 min/d, for 2 and 3 wk.	N/A	LIPUS increased proliferation, GAG, Col II, and total collagen. LIPUS stimulation could be a highly efficient tool for tissue engineering in combination with 3D printing and hMSCs to regenerate damaged cartilage tissues.	(52)
29,689,491	Chen et al. (2018)	N/A	Male New Zealand white rabbits osteochondral defects OA model.	The hybrid use of growth factors and LIPUS stimulation exhibited good potential in enhancing vascularization and the formation of new bone and cartilage, providing better conditions for osteochondral repair.	(55)
31,941,483	Nasb et al. (2020)	HADMSCs. 0.5 W/cm^2^, 1 MHz, 20 min/d, 5d/wk., for 8 wk.	N/A	The primary outcome is the Western Ontario and McMaster Universities Index of OA (WOMAC) score, while the secondary outcomes will be other knee structural changes and lower limb muscle strength, such as the knee cartilage thickness measured by MRI. Blinded assessments will be performed at baseline (1 month prior to treatment), 1 month, 3 months, and 6 months following the interventions.	(47)
32,547,027	Chen et al. (2020)	Human primary OA-chondrocytes. 0.5 W/cm^2^, 1.0 MHz, 20 min/d at each other day for 7d.	Male spontaneous OA Dunkin-Hartley guinea pigs. 0.1 W/cm^2^, 3 MHz, 20 min/d, thrice a week (once every other day) for 8 wk.	L-rapa combined with LIPUS possessed the most consistent and effective anabolic and anti-catabolic effects in HOACs and spontaneous OA guinea pigs. This study revealed that liposome-encapsulation collaborated with LIPUS can reduce the effective dose and administration frequency of rapamycin and further stably reinforce its therapeutic actions against OA.	(56)
33,412,895	Xia et al. (2021)	rBMSCs. 20, 30, 40, and 50 mW/cm^2^, 3 MHz, 20 min/d, for 10 d.	Male SD rats ACLT-OA model. 20, 30, 40, and 50 mW/cm^2^, 3 MHz, 20 min/d, once every 10 days for 4 times	LIPUS enhanced the migration of MSCs through the activation of autophagy, and LIPUS improved the protective effect of MSCs on OA cartilage via autophagy regulation.	(48)
34,102,487	Liao et al. (2021)	rBMSCs. 30 mW/cm2, 1.5 MHz, 20 min.	Male SD rats ACLT&MMx-OA model. 30 mW/cm^2^, 1.5 MHz, 20 min/d, for 4 times	LIPUS-mediated BMSC-derived exosomes promoted cartilage regeneration, increased chondrocyte proliferation and extracellular matrix synthesis, suppressed inflammation, and inhibited the IL-1β-induced activation of the NF-κB pathway.	(49)
34,149,994	Zuo et al. (2021)	Rabbit primary chondrocytes. 30, 45, and 60 mW/cm^2^, 3 MHz, 20 min.	Male New Zealand rabbits ACLT-OA model. 60 mW/cm^2^, 1.5 MHz, 20 min/d, 5 d/wk. for 6 wk.	Combined PBNPs/LIPUS treatment, which was superior to either PBNPs or LIPUS in articular cartilage protection, could alleviate ROS and apoptosis of chondrocytes by activating the PI3K/Akt/mTOR pathway, reduce inflammatory cytokines and inhibit ECM degradation by reducing MMPs expression.	(57)
35,438,034	Xia et al. (2022)	rBMSCs and human primary OA chondrocytes. 50 mW/cm^2^, 3 MHz, 20 min/d, for 0, 3, 7, and 10 d.	Male SD rats ACLT-OA model. 50 mW/cm^2^, 3 MHz, 20 min/d, for 4 wk.	LIPUS promoted MSC exosome release by activating autophagy and significantly enhanced the positive effects of MSCs on OA cartilage by blocking GW4869, an inhibitor of exosome release.	(39)

ECM, extracellular matrix; PI3K, phosphatidylinositol 3-kinase; Akt (PKB), protein kinase B; MAPK, mitogen-activated protein kinases; TD, dexamethasone/transforming growth factor-beta1; ACAN, aggrecan; BMP-2, bone morphogenetic protein-2; ALP, alkaline phosphatase; Runx2, runt-related transcription factor 2; MSC, mesenchymal stem cell; BV/TV, bone volume per tissue volume; GAG, glycosaminoglycan; HADMSCs, human adipose-derived mesenchymal stem cells; L-rapa, liposome-encapsulated rapamycin; NF-κB, nuclear factor-k-gene binding; PBNPs, Prussian blue nanoparticles; ROS, reactive oxygen species; PI3K, phosphatidylinositol-3-kinase; mTOR, mammalian target of rapamycin.

### 4.1 Seed cells

Selecting the appropriate seed cells is imperative in the development of an optimal tissue-engineered articular cartilage. The chosen cells must possess robust capacities for proliferation and differentiation, enabling them to mature while preserving the chondrocyte phenotype. Mesenchymal stem cells are a favorable choice for seed cell selection in tissue engineering applications due to their accessibility from multiple sources, such as bone marrow, adipose tissue, periosteum, synovial fluid, dermis and blood, all of which provide substantial potential for proliferation and differentiation.

Nasb et al. (47) conducted a study with 96 OA patients, who were divided into three groups in a 1: 1:1 ratio to receive intraarticular injections of human adipose-derived mesenchymal stem cells (HADMSCs) with LIPUS, HADMSCs with sham LIPUS, or normal saline with LIPUS. They observed that LIPUS enhanced the tissue repair function in MSCs. Xia et al. (48) discovered that LIPUS increased the expression of Stromal cell-derived factor-1 (SDF-1) and C-X-C chemokine receptor type 4 (CXCR4) proteins, thereby promoting MSC migration. They also found that autophagy inhibitors and agonists could influence this effect. The findings from *in vivo* experiments revealed that LIPUS significantly enhanced the therapeutic effects of MSCs on OA by promoting cartilage repair. This effect was found to be modulated by specific inhibitors. Through the activation of autophagy, LIPUS facilitated the migration of MSC and strengthened their protective effects on OA cartilage. In a study by Yamaguchi et al. (43), the combined treatment of LIPUS and MSC injection was shown to improve cartilage repair and promote subchondral bone reconstruction in osteochondral defects. Additionally, MSC injection was found to enhance cartilage repair scores, while LIPUS irradiation increased BV/TV. The synergistic effects of combined treatment involving MSC injection and LIPUS irradiation have been shown to enhance cartilage repair and BV/TV improvement. This combined approach is more effective in promoting concurrent cartilage repair and subchondral reconstruction compared to either treatment alone. Furthermore, LIPUS has been found to augment the regenerative effects of BMSC-derived exosomes on OA cartilage by suppressing inflammation, stimulating chondrocyte proliferation, and enhancing cartilage matrix synthesis, potentially through the inhibition of the NF-κB pathway activation induced by IL-1β (49).

### 4.2 Scaffolds

Scaffolds play a role in cartilage tissue engineering by offering a structure framework for the adhesion, growth, and specialization of seed cells, necessitating characteristics such as biocompatibility, biodegradability, and biomechanical stability. Biocompatibility ensures the scaffold’s compatibility with the surrounding biological milieu, while biodegradability facilitates gradual decomposition as new tissue develops. Furthermore, it is imperative for the biomechanical properties of the scaffold to closely resemble those of natural cartilage in order to facilitate structural support during the process of regeneration. Consequently, scaffolds serve as a conducive environment for seed cells to flourish, thereby promoting the synthesis of regenerated hyaline cartilage and facilitating the successful repair of cartilage defects.

The utilization of three-dimensional (3D) printing technology and ultrasound therapy has garnered significant interest in the field of cartilage tissue engineering. Takeuchi et al. (50) employed type-I honeycomb collagen sponges as a 3D scaffold for chondrocyte culture, observing enhanced chondrocyte proliferation and Col IX synthesis following LIPUS stimulation via the integrin/PI3K/Akt signaling pathway. Uenaka et al. (51) developed a semi-open static culture system to enhance the generation of scaffold-free constructs from monolayer-cultured chondrocytes, with the additional stimulation of ECM production through LIPUS. Aliabouzar et al. (52) investigated the seeding of human mesenchymal stem cells (hMSCs) on 3D printed poly-(ethylene glycol)-diacrylate (PEG-DA) scaffolds with varying pore sizes. Their findings indicated that square porous scaffolds exhibited superior effects on cell proliferation and chondrogenic differentiation compared to solid or hexagonal scaffolds, showing a significant increase in the production of GAG, Col II, and other anabolic products after 24 h of LIPUS stimulation. Researchers combined LIPUS with lip-coated microbubbles to improve ultrasound imaging and drug delivery, as well as to stimulate cell growth and chondrogenic differentiation (53). Babaei et al. (54) cultured chondrocytes on a 3D printed polyurethane scaffold infused with gelled glue, hyaluronic acid and glucosamine, and observed that LIPUS treatment enhanced cell proliferation, viability, and gene expression related to chondrocyte phenotype. Chen et al. (55) conducted optimization and design a single-layer integrated multi-layer functional structure functional bionic scaffold for the repair of osteochondral defects, utilizing rabbit model experiments to evaluate the efficacy of the repair. The findings indicated successful integration at the interface of the scaffold material and host tissue, as well as between the newly formed subchondral bone and cartilage. In this study, osteochondral repair demonstrated the highest total histological score. The experimental model involved the use of cylindrical fully repaired full-thickness defects measuring 5 mm in diameter in rabbits. Of particular note is the similarity in thickness between the regenerated cartilage and the surrounding normal cartilage, as well as the resemblance in cell arrangement and number the superficial cartilage region to that of normal hyaline cartilage. Additionally, distinct lacunae were observed in the regenerated cartilage.

### 4.3 Growth stimulating factors

Growth-stimulating factors are crucial in facilitating and enhancing the tissue engineering process. With the concurrent application of these factors with LIPUS stimulation has shown promising results in enhancing vascularization and facilitating the development of new bone and cartilage, thereby creating a conductive environment for comprehensive mitochondrial repair (55). Chen et al. (56) conducted a study in which drug carriers were substituted with liposomes to prolong drug effectiveness and decrease possible adverse effects, resulting in decreased effective dosage and frequency of administration for OA repair through the concurrent use of LIPUS. This approach ultimately improved the consistency of therapeutic outcomes. Additionally, Zuo et al. (57) found that the combination of LIPUS with Prussian blue nanoparticles (PBNPs) led to reduced cellular reactive oxygen species (ROS), apoptosis, and MMPs, thereby preventing articular cartilage damage via the PI3K/Akt/mTOR signaling pathway. The utilization of dexamethasone and transforming growth factor-b1 (TD) in the treatment of hMSCs resulted in the manifestation of characteristic cartilage cell morphology. The incorporation of LIPUS further increased the effects of TD on the differentiation of hMSCs (58). Although several growth-stimulating factors have shown efficacy in promoting cell proliferation and differentiation *in vitro*, the suitability of these stimuli necessitates comprehensive validation and careful selection.

## 5 Perspectives, future outlook and remarks

Originally employed for the management of delayed fracture union and non-union cases, the utilization of LIPUS has evolved to include a wide range of applications in the fields of musculoskeletal, neurological, urinary, cardiovascular, and other systemic disorders. According to the mechano-biochemical transduction theory, LIPUS impact cellular function through the induction of cavitation and mechanical responses within various cellular components, including the extracellular matrix, cell membrane, cytoplasm, and nucleus, via mechanisms involved in structural integrity and motility. This ultimately results in a range of biological effects, including alterations in cellular morphology, regulation of gene expression, and metabolic adaptations. Furthermore, LIPUS enhances the synthesis of crucial factors (90, 91) essential for cellular proliferation and tissue regeneration, such as TGF, FGF, VEGF, and BMP. Collectively, these intricate biological mechanisms suggest that LIPUS holds potential as a therapeutic intervention for conditions such as OA.

Currently, numerous *in vitro* research provides evidence for the efficacy of LIPUS as a non-invasive therapeutic intervention for modulating synthesis/catabolism levels in OA joints and facilitating changes in the synovial membrane, cartilage, and subchondral skeletal microenvironment. Given the avascular nature of cartilage and the hypoxic conditions (92–94) in which chondrocytes function intra-articularly, the majority of *in vitro* and *ex vivo* studies have been conducted under standard 21% O_2_ and 5% CO_2_ concentrations. The heightened oxygen levels present in the incubator exceed the physiological levels found in live cartilage potentially leading to alterations in chondrocyte response to LIPUS and subsequent biochemical changes. Additionally, it is crucial to consider that many *in vitro* studies have not yet investigated the potential influence of cell culture materials and media on the transmission of LIPUS waves. Nevertheless, the intrinsic attributes of these cultural elements possess the capacity to affect the transmission of LIPUS waves, thereby significantly influencing cellular biochemical responses. Thus, a comprehensive comprehension of the synergistic effects of LIPUS and the extracellular milieu is imperative for the development of a culture system that more accurately replicates the intracellular microenvironment.

It is imperative to perform preclinical studies utilizing appropriate animal models in order to investigate the effects of LIPUS on the entire joint and its potential chondroprotective properties. Risk factors associated with OA can be classified into mechanical and non-mechanical factors, each leading to unique manifestations of the disease. Synovial inflammation persists throughout the progression of OA, while cartilage degeneration is a prominent pathological characteristic in the intermediate and advanced stages. The initial phases of OA involve the activation of osteoclasts in the subchondral bone, progressing to osteoporosis in advanced stages. Given the differences in load-bearing capacities between various animal species and human joints, careful consideration must be given to the selection of appropriate experimental animals for both surgical and non-surgical modeling at different stages OA development.

LIPUS is characterized by acoustic radiation whose power diminishes as a function of distance. Upon encountering tissues within a joint, LIPUS undergoes reflection and refraction, thereby modifying the mechano-biochemical transduction effects on various tissues, cells, and molecules. The efficacy of LIPUS interventions in activating molecular pathways and cellular activities is contingent upon factors such as duration, strength, and energy parameters, which may vary across studies, posing challenges in the analysis and comparison of results. Additionally, it is important to note that treatment outcomes may differ depending on the specific LIPUS machines, probe types, intervention techniques, and individual variations. Therefore, there is a critical necessity for additional high-quality evidence to substantiate its effectiveness. In the development of LIPUS devices for both animal and clinical trials, medical researchers should engage in collaboration with professionals in acoustic engineering, software engineering and AI algorithms to guarantee the creation of experimental instruments capable of managing acoustic peak pressure, radiation pressure, and acoustic flow force, thereby accurately controlling the LIPUS intensity.

Tissue engineering, specifically utilizing LIPUS, has experienced notable progress and is widely utilized for joint repair. Nevertheless, there are several persistent challenges. One such challenge is the diminished differentiation potential of mesenchymal stem cells with age, coupled with limitations in the quantity of cells that can be obtained from these stem cells due to the difficulty in maintaining their chondrocyte phenotype post-differentiation. Consequently, there is a potential for hypertrophic differentiation in these cells, which could result in apoptosis or ossification. Moreover, the precise cartilage subtype that may be generated remains uncertain. The field of materials science has experienced significant progress, facilitating the discovery and application of contemporary natural and synthetic materials for potential use as scaffold materials in tissue engineering. Through the utilization of current theoretical models and methodologies, the processes and outcomes of tissue engineering have undergone continuous enhancement. Techniques such as 3D cell culture *in vitro* epitaxy and 3D bioprinting have played a crucial role in the development of cartilage tissues with improved properties. However, additional validation studies and clinical trials are necessary to confirm their practical advantages. Growth factors, including TGF, BMP, and others, are thought to promote the growth and specialization of precursor cells *in vitro*. Despite encouraging preliminary results, the optimization and selection of these stimulating factors require further exploration for practical implementation.

The current state of clinical application of LIPUS therapy is characterized by a complex interplay of potential benefits, challenges, and limitations. These include the high cost of LIPUS treatment devices, which restricts accessibility to a wider patient demographic, variability in treatment outcomes leading to inconsistent efficacy, and the time-intensive nature of the therapy requiring multiple visits over an extended duration, potentially impacting patient compliance and quality of life. While LIPUS treatment is commonly perceived as noninvasive and safe, certain patients may encounter adverse effects such as skin irritation or discomfort. Furthermore, the efficacy of this therapy is constrained by the specific characteristics and site of the injury or illness, thereby diminishing its overall effectiveness. Lastly, the utilization of LIPUS therapy necessitates healthcare professionals to undergo specialized training for its appropriate administration, consequently augmenting the intricacy and expenses associated with delivering this treatment. In summary, LIPUS presents numerous advantages compared to traditional pharmacological treatments, including its localized application, non-invasiveness, ease of use, cost-effectiveness, and efficacy. As further research is conducted, LIPUS is poised to significantly enhance the management of OA for a large population of patients globally. It is essential to acknowledge that despite the promising results of LIPUS therapy in specific clinical contexts, its widespread acceptance and implementation are contingent upon addressing substantial obstacles and constraints.

## Author contributions

JZ: Formal analysis, Writing – review & editing, Writing – original draft. EN: Data curation, Writing – review & editing. LL: Data curation, Writing – review & editing. HZ: Data curation, Writing – review & editing. XY: Conceptualization, Supervision, Writing – review & editing. YH: Conceptualization, Supervision, Writing – review & editing.
